# Peptides presenting the binding site of human CD4 for the HIV-1 envelope glycoprotein gp120

**DOI:** 10.3762/bjoc.8.214

**Published:** 2012-10-31

**Authors:** Julia Meier, Kristin Kassler, Heinrich Sticht, Jutta Eichler

**Affiliations:** 1 Department of Chemistry and Pharmacy, Universität Erlangen-Nürnberg, Schuhstrasse 19, 91052 Erlangen, Germany; 2 Institute of Biochemistry, Universität Erlangen-Nürnberg, Fahrstrasse 17, 91054 Erlangen, Germany

**Keywords:** biomimetic synthesis, CD4, HIV entry, peptide, protein binding site

## Abstract

Based on the structure of the HIV-1 glycoprotein gp120 in complex with its cellular receptor CD4, we have designed and synthesized peptides that mimic the binding site of CD4 for gp120. The ability of these peptides to bind to gp120 can be strongly enhanced by increasing their conformational stability through cyclization, as evidenced by binding assays, as well as through molecular-dynamics simulations of peptide–gp120 complexes. The specificity of the peptide–gp120 interaction was demonstrated by using peptide variants, in which key residues for the interaction with gp120 were replaced by alanine or D-amino acids.

## Introduction

Synthetic molecules that have the ability to mimic binding and/or functional sites of proteins are useful tools for exploring and modulating protein function, as they interfere with binding events underlying the protein function. Furthermore, such mimetic molecules are promising candidates for the inhibition of protein–protein interactions. Synthetic peptides can be produced as direct reproductions of protein fragments and by diverse chemical modification, including the integration of non-proteinogenic amino acids, and the modification of the peptide backbone. Such modifications widen the chemical and structural diversity exhibited by peptides, as well as improve their proteolytic stability, increasing their prospects for pharmaceutical use. Therefore, peptides are excellent candidates as protein binding site mimics. We have previously developed strategies for the design and generation of scaffolded and assembled peptides to generate protein binding site mimics [1].

The interaction of the HIV-1 envelope glycoprotein gp120 with its cellular receptor CD4 is the first step in the process of entry of the virus HIV-1 into its host cell. A range of crystal structures of the gp120–CD4 complex [2–6] have provided information on the structural details of the gp120–CD4 interaction and, thus, the basis for a rational design of inhibitors. Later on, it was found that the epitopes of a range of broadly neutralizing anti-HIV-1 antibodies, such as mAb b12 [7] and mAb VRC01 [8], overlap the CD4 binding site (CD4bs) of gp120, making this region of gp120 an important target for immunogen design. Therefore, we have previously designed and generated a synthetic peptide that presents the CD4bs fragments of gp120 [9]. This peptide is recognized by CD4, as well as by mAbs b12 and VRC01.

**Figure 1:**
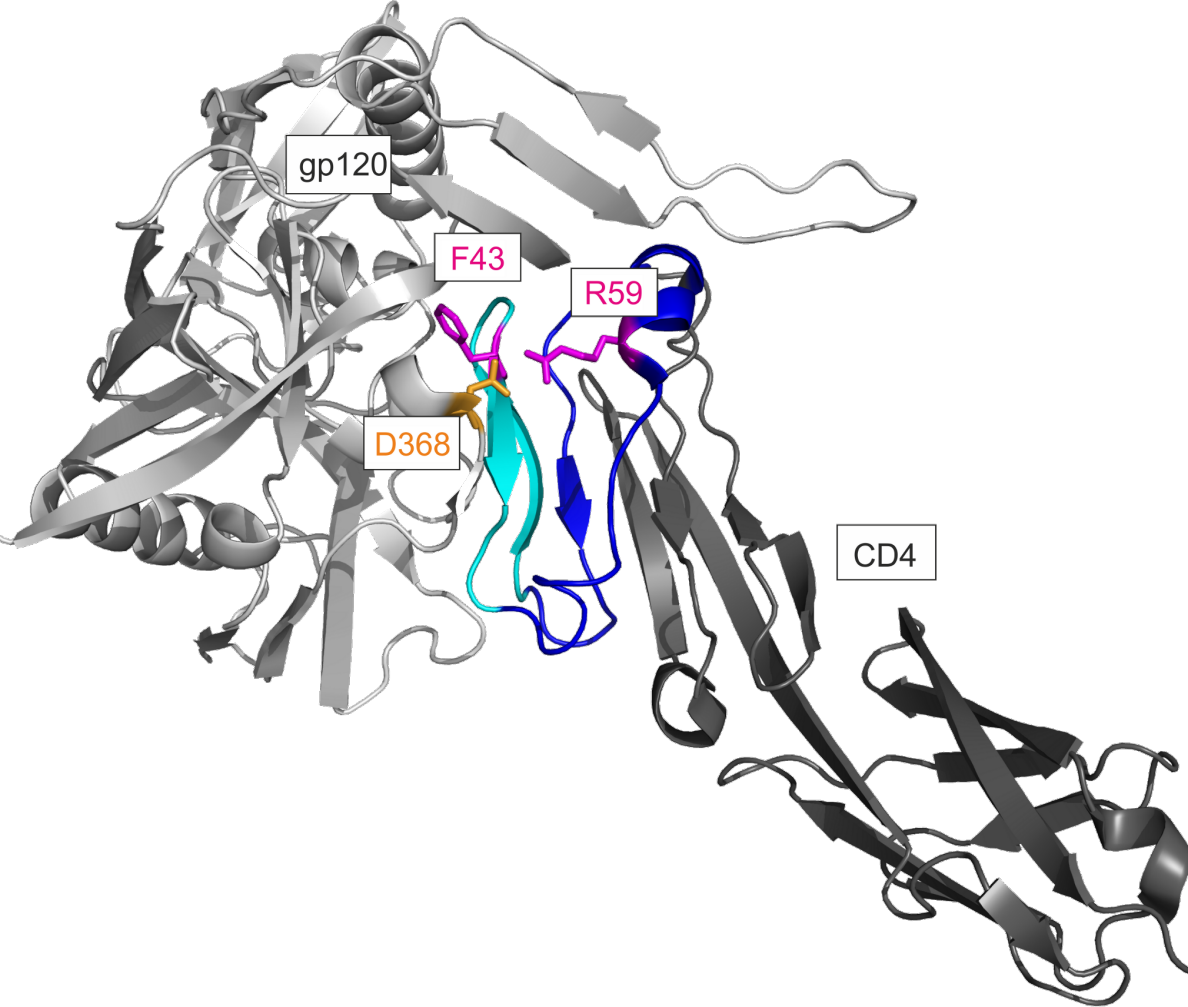
Structural details of the CD4–gp120 complex (pdb 1rzj). The binding site of CD4 for gp120 is shown in blue (residues 22–33 and 49–64) and in cyan (CDR2-like loop, residues 34–48). The key residues for the interaction with gp120 (F43 and R59) are depicted in purple, and the contact residue D368 of gp120 in yellow.

The receptor glycoprotein CD4 is composed of four extracellular immunoglobulin domains (D1–D4), a short cytoplasmatic tail, and a single transmembrane helix [10]. Gp120 contacts the CD4 D1 domain, which forms a stable eight-stranded beta-sheet [3] (Figure 1). The 22 residues of CD4 that contact 26 amino acids of gp120 [3] are located in the N-terminus of CD4 D1 (residues 22–64). Hot spots of CD4 for its interaction with gp120 include F43 at the tip of the CDR2-like loop, which contacts the CD4 binding site of gp120. Ionic interactions of R59 of CD4 with D368 of gp120 stabilize this interaction [3] (Figure 1). Mutation of F43 and R59 to alanine or glycine [11–14] dramatically impairs binding to gp120, corroborating the importance of F43 and R59 for the interaction of CD4 with gp120.

Molecules that are capable of inhibiting the CD4–gp120 interaction are promising candidates for HIV-1-entry inhibitors, an upcoming class of AIDS therapeutics that offer an alternative to the clinically established anti-HIV-1 drugs, which are mainly inhibitors of HIV-1-encoded enzymes (protease, reverse transcriptase and integrase). During recent years, a range of structurally different CD4 mimetic molecules have been presented. These include small molecules [15–18] as well as stably folded miniproteins, which were mutated to present a putative binding site for gp120 [19–21].

We have recently shown that synthetic peptides mimicking the gp120 binding sites of human and murine CD4 can be used as molecular tools to elucidate the structural basis for the species selectivity of the CD4–gp120 interaction [22]. We have now focused on peptides mimicking the binding site of human CD4, and on their interaction with gp120.

## Results and Discussion

### Peptide design and synthesis

**Figure 2:**
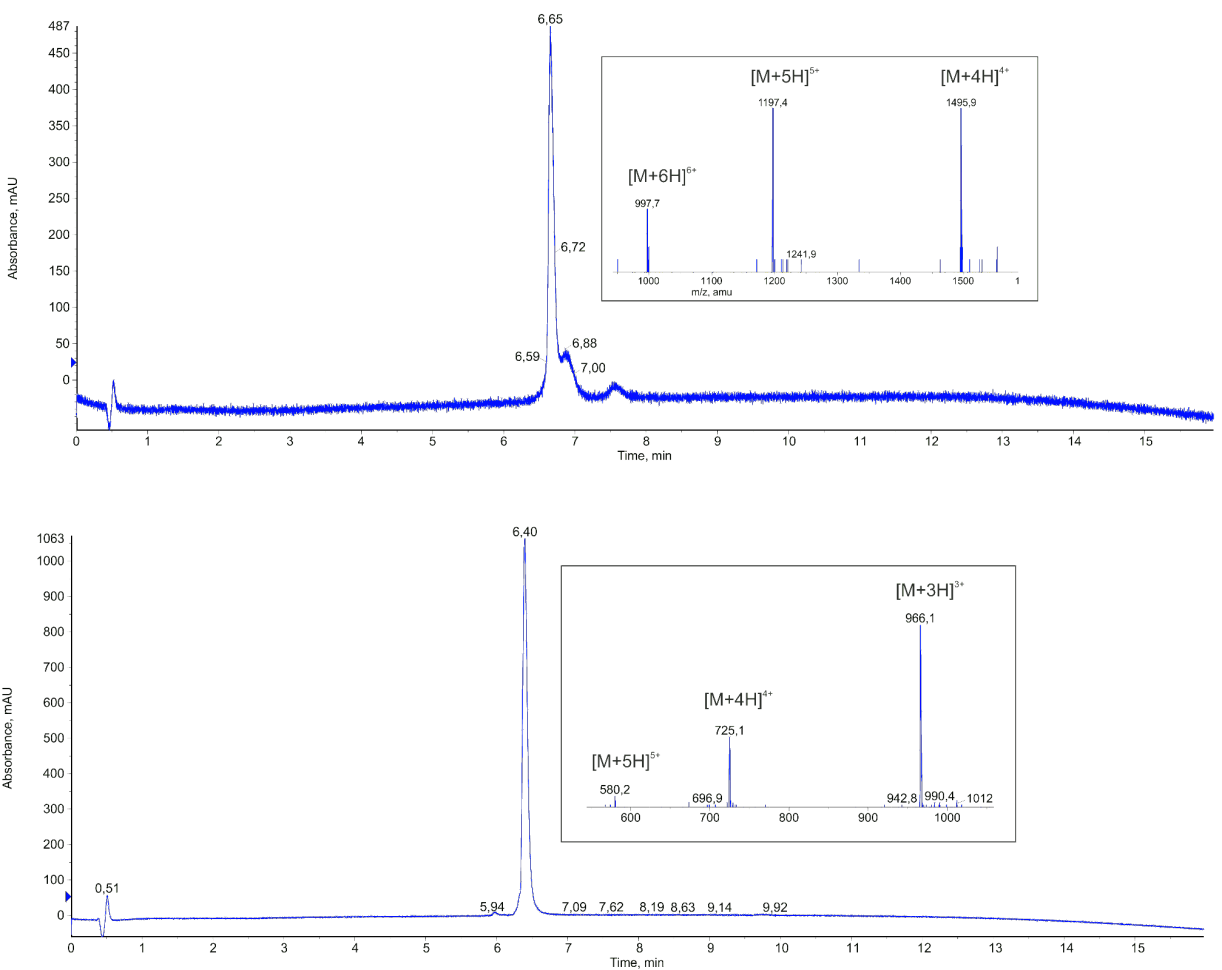
HPLC chromatograms and ion masses from the ESI-mass spectra (insets) of purified CD4-M2 (M = 5979.83, top) and CD4-M5 (M = 2896.4, bottom).

Based on the resolved 3D structure of the gp120–CD4 complex, we have designed peptides that present the binding site of CD4 for gp120, i.e., residues 22–64 (Table 1). Apart from the peptides covering this complete CD4 stretch (CD4-M1), we generated a truncated peptide presenting only the CDR2-like loop of CD4 (residues 34–48, CD4-M4). Since the N- and C-termini of CD4-M1 and CD4-M4 are in fairly close proximity in the CD4-gp120 complex [3], as evidenced by the short distances between S23 and D63 (4.6 Å) as well as between I34 and P48 (10.6 Å), we also generated peptides in which this proximity is covalently stabilized. This was achieved by means of a disulfide bridge between cysteine residues, which were introduced either by replacing S23 and D63 with cysteine (CD4-M2 and CD4-M3), or by being added to either side of the CD4 sequence (CD4-M5, CD4-M6 and CD4-M7). Furthermore, variants CD4-M2 and CD4-M5 were synthesized in which the key amino acids for the interaction with gp120 (F43 and R59) were replaced by alanine (CD4-M3 and CD4-M6). In CD4-M3, we also replaced the adjacent R58 by alanine in order to avoid functional compensation for the loss of R59 by R58. Finally, we probed the stereoselectivity of the peptide–gp120 interaction by replacing F43 in CD4-M5 by D-phenylalanine (CD4-M7). All peptides were equipped with an N-terminal hexahistidine tag for directed attachment to Ni-NTA assay plates. The tag, as well as the additional cysteine residues in CD4-M5, CD4-M6 and CD4-M7, was separated from the CD4 sequences by the spacer amino acids ε-aminohexanoic acid (X) and β-alanine (B). All peptides were generated through solid-phase synthesis and purified by preparative HPLC (Figure 2 and Experimental section).

**Table 1:** Peptide sequences.

Peptide	CD4-residues	Sequence

CD4-M1	22–64, linear	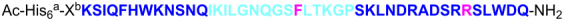
CD4-M2	22–64, cyclic, S23C, D63C	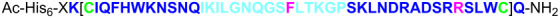
CD4-M3	22–64, cyclic, F43A, R58A, R59A, S23C, D63C	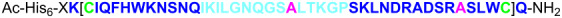
CD4-M4	34–48, linear	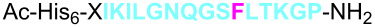
CD4-M5	34–48, cyclic	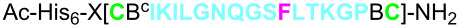
CD4-M6	34–48, cyclic, F43A	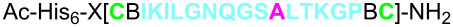
CD4-M7	34–48, cyclic, F43-D-Phe	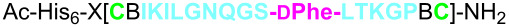

^a^His_6_, hexahistidine tag; ^b^X, ε-aminohexanoic acid; ^c^B, β-alanine. Brackets indicate a disulfide bridge between cysteine residues. All residues that are part of CD4 are shown in bold face and blue (residues 22–33 and 49–64) or cyan (CDR2-like loop). The hot spot residues F43 and R59, or their substitutes, are shown in red.

### Peptide binding to gp120

**Figure 3:**
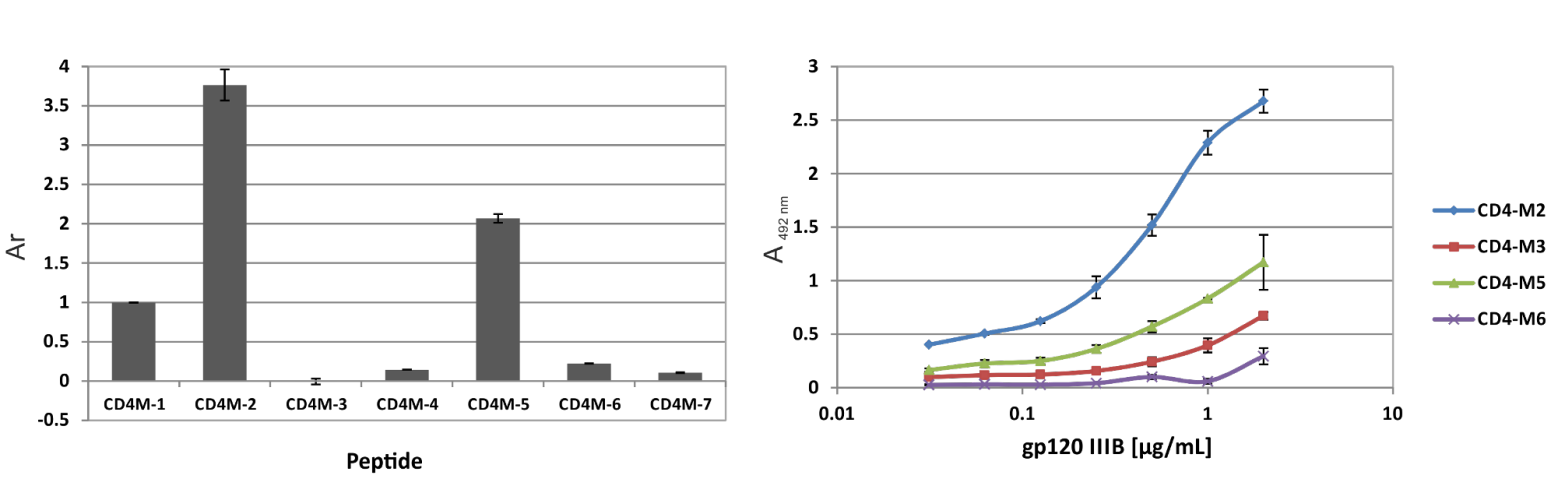
Left: Relative affinities (*A*_r_, CD4-M1 = 1) to gp120(IIIB) of peptides. Right: Concentration-dependent binding of gp120 to peptides. Absorbance values (A) are corrected for the respective blanks (wells without peptide). See Table 1 for peptide sequences and Experimental section for details of the binding assay.

The seven CD4-derived peptides (Table 1) were tested in an ELISA type binding assay for their ability to bind to recombinant gp120 from HIV-1 strain IIIB (Figure 3, left). Of the two linear peptides (CD4-M1 and CD4-M4), only the longer peptide CD4-M1 is recognized by gp120, illustrating the importance of the salt bridge R59 for the stabilization of the interaction of the CDR2-like loop of CD4 with gp120, as CD4-M4 lacks this residue. Covalent stabilization of the spatial proximity between the N- and C-termini of peptides CD4-M1 and CD4-M4 through a disulfide bridge in the cyclic peptides CD4-M2 and CD4-M5 appears to stabilize the gp120-bound conformation, since binding of both peptides to gp120 was strongly enhanced, as compared to their linear, more flexible counterparts. The specificity of this interaction could be demonstrated by using analogues of CD4-M2 and CD4-M5, in which the key residues for the interaction with gp120, (F43 and R59), were replaced by alanine (CD4-M3 and CD4-M6, respectively). Binding of these alanine variants to gp120 was largely abrogated, indicating that the mode of binding of CD4-M2 and CD4-M5 to gp120 is related to that of CD4. Likewise, an analogue of CD4-M5, in which F43 was replaced by D-phenylalanine (CD4-M7), did not bind to gp120, most likely because the incorrect orientation of the phenyl side chain of this amino acid precludes its interaction with D368 and E370 of gp120, which are key residues of the CD4 binding site of gp120. The differences in affinity to gp120 between the cyclic peptides CD4-M2 and CD4-M5 on one hand, and their alanine substitution variants CD4-M3 and CD4-M6 on the other hand, could also be shown in a more quantitative fashion by dose-dependent binding of gp120 (Figure 3, left).

**Figure 4:**
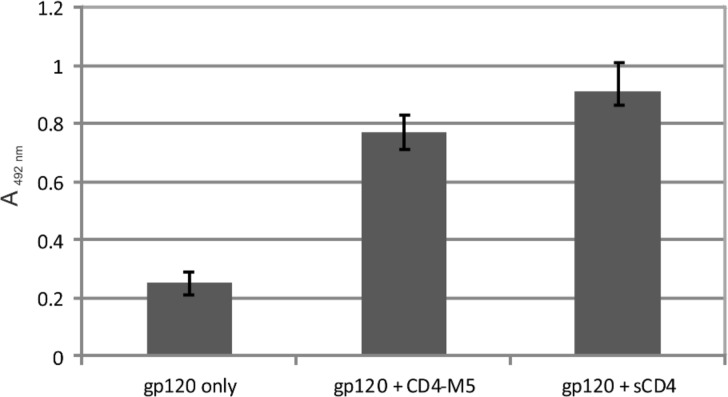
Enhancement of binding of gp120(MN) to the CD4i antibody X5 by CD4-M5 and sCD4, respectively. See Experimental section for details of the binding assay.

In a separate binding assay, we addressed the interaction of HIV-1 with cellular coreceptors. This interaction is induced by binding of gp120 to cellular CD4, which triggers a conformational rearrangement of gp120, resulting in the exposure of its coreceptor binding site [23]. Such binding enhancement could also be demonstrated in binding assays involving recombinant proteins, i.e., gp120, and soluble CD4 (sCD4) presenting the extracellular CD4 domains, in conjunction with the use of antibodies that recognize the coreceptor binding site of gp120 (CD4i antibodies) as coreceptor surrogates [8]. Similar to sCD4, the cyclic peptide CD4-M5, which presents the CDR2-like loop of CD4, is able to enhance binding of gp120 to the CD4i antibody mAb X5 [24] (Figure 4), providing further indication of a functional mimicry of CD4 by the mimetic peptides.

### Comformational stability of gp120-peptide complexes

**Figure 5:**
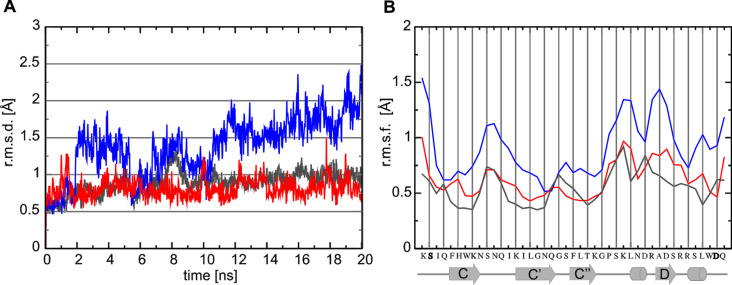
Conformational stability of CD4 mimetic peptides in complex with gp120 during 20 ns of MD simulation. Results are shown in blue (CD4-M1) and red (CD4-M2), respectively. For comparison, the respective sequence region has also been analysed for the CD4–gp120 complex (black). (A) Root-mean-square deviation of the peptides and CD4 over simulation time. (B) Root-mean-square fluctuation of individual residues measured for backbone atoms averaged over time. Residues replaced by cysteine in CD4-M2 are indicated in bold face. Secondary structure elements are assigned below the plot.

To investigate the conformational stability and binding properties of CD4-M1 and CD4-M2, molecular dynamics (MD) simulations of the peptides in complex with gp120 were carried out and compared to a simulation of the CD4–gp120 complex. Analysis confirmed that the linear peptide CD4-M1 is more flexible than the respective region in CD4 (Figure 5A) because it lacks the stabilizing interactions contributed by the remaining parts of intact CD4 (Figure 1).

**Figure 6:**
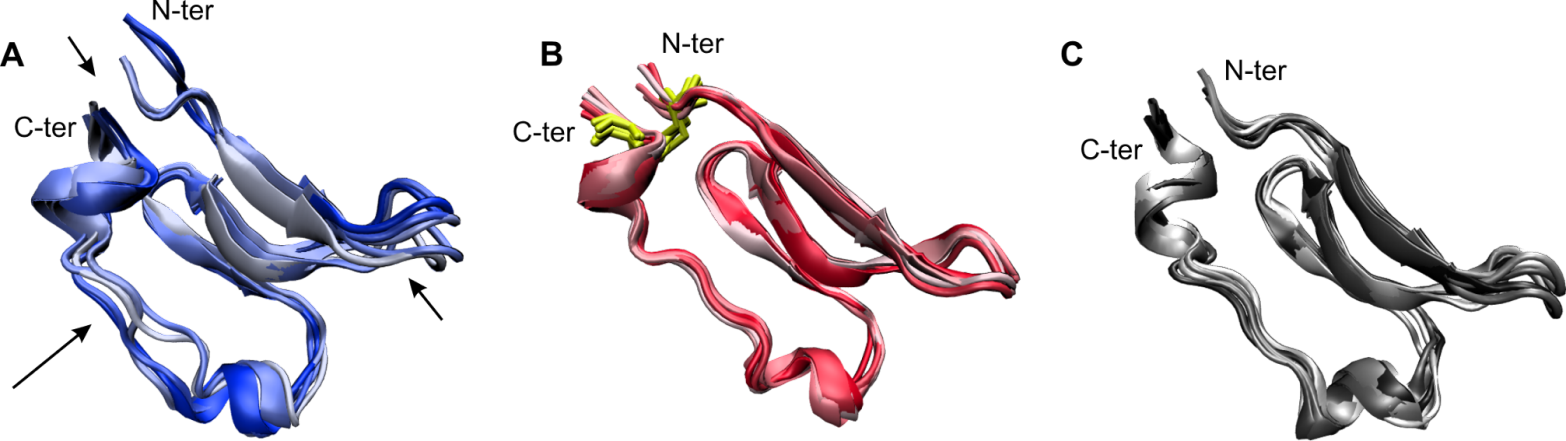
Structural presentation of peptide motions in the gp120-bound state during the MD simulation. (A) CD4-M1, (B) CD4-M2, (C) respective sequence stretch of CD4 (the remaining part of CD4 has been omitted for simplicity in this plot). The disulfide bridge that fixes N- and C-termini in CD4-M2 is represented by yellow sections. Arrows indicate regions in CD4-M1 that show enhanced fluctuation during simulation, namely the N- and C-termini, the CC’-loop and the stretch between the short 3_10_-helixes upstream of the C-terminus.

Interestingly, the introduction of a disulfide bridge between C23 and C63 in CD4-M2 re-establishes the rigidity present in the CD4–gp120 complex. This is evident from the conformational stability shown in Figure 5A and the per-residue fluctuations shown in Figure 5B. In both types of analysis, the cyclic peptide CD4-M2 is significantly more stable than the linear peptide CD4-M1, with a conformational stability similar to the respective region in CD4. The larger flexibility of CD4-M1 also becomes evident from a visual analysis of the structures over time (Figure 6). Regions of enhanced fluctuation comprise the CC’-loop, both termini, and the C-terminal stretch containing two short 3_10_-helices (Figure 5B and Figure 6A). Hence, it appears that the introduction of a disulfide bridge between the N- and C-terminus in CD4-M2 stabilizes not only the termini but also other flexible regions of the peptide, suggesting a global stabilization of the peptide (Figure 6B). The overlays shown in Figure 6B and Figure 6C again demonstrate that the conformational rigidity of gp120-bound CD4-M2 is similar to that of the respective region in CD4.

Another observation from the binding assay was the complete loss of binding of the triple alanine mutants (F43A, R58A, R59A) of CD4-M2 (peptide CD4-M3). Analysis of the interactions formed by these residues over the simulation time shows that F43 and R59 preserve the stable interactions with gp120 that are present in the gp120–CD4 complex (Figure 1), while R58 does not significantly contribute to binding in any of the simulations. The importance of F43 and R59 for peptide binding also supports the notion that both peptides bind specifically to the CD4 binding site of gp120.

## Conclusion

The binding behaviour of synthetic CD4 mimetic peptides was shown to be strongly related to their conformational flexibility, as demonstrated by binding assays in conjunction with molecular-dynamics simulations. These results illustrate the utility of peptide mimics of protein binding sites as molecular tools to explore the molecular and structural basis of protein–protein interactions. Furthermore, this strategy may be potentially useful for the structure-based design of synthetic protein-binding-site mimics by improving the conformational stability of the mimetic peptides, which in turn will increase their affinity to the complementary protein, and, consequently, their ability to interfere with the native protein–protein interaction.

## Experimental

### Peptide synthesis

Peptides were synthesized as C-terminal amides by Fmoc/*t*-Bu-based solid-phase synthesis on 100 mg TentaGel S RAM resin (0.23 mmol/g) by using an automated multiple peptide synthesizer (SYRO from MultiSynTech), as previously described [25]. In a standard coupling cycle, five equiv of Fmoc-amino acid/*N*,*N*’-diisopropylcarbodiimide/*N*-hydroxybenzotrialzole in DMF were coupled twice for 60 min, followed by a capping step using a mixture of acetic anhydride/pyridine/DMF (1:2:3; 30 min). The Fmoc group was removed by using 20% piperidine/DMF (20 min). After completing the sequence, the N-terminal amino group was acetylated. Peptides were cleaved from the resin by using Reagent K (TFA/water/phenol/thioanisole/1,2-ethanedithiol; 82.5:5:5:5:2.5), precipitated in a cold 1:1 mixture of cyclohexane and tert-butyl methyl ether, extracted with water, lyophilized twice, and purified by preparative HPLC (conditions: column: Dr. Maisch Reprosil 100, 250 × 20 mm, flow rate: 9 mL/min, gradient: 35–45% ACN in H_2_O (both containing 0.1% TFA) in 40 min and UV detection at 216 nm). Disulfide bridges were formed by oxidizing the peptides (0.25 mg/mL in 50% ACN in 0.1 M ammonium acetate, pH 8) at room temperature with slight shaking until free thiol groups were no longer detectable by Ellman’s reagent. Oxidized peptides were purified again as described above. Peptides were characterized by analytical HPLC with online ESI mass spectrometry detection (LC–MS). Conditions: column: Phenomenex Kinetex 2.6 µM C18 100 Å, 50 × 2.1 mm, flow rate: 0.4 mL/min, gradient: 5–95% ACN in H_2_O (both containing 0.05% TFA) in 15 min (see Table 2 for mass spectrometry data).

**Table 2:** ESI-mass spectrometry data of peptides.

Peptide	M_calc_	[M + 2H]^2+^	[M + 3H]^3+^	[M + 4H]^4+^	[M + 5H]^5+^	[M + 6H]^6+^	[M + 7H]^7+^	[M + 8H]^8+^

CD4-M1	5977.72			1494.9	1196.0	996.8	854.3	748.1
CD4-M2	5979.83			1495.9	1197.4	997.7	855.3	748.6
CD4-M3	5733.51			1433.5	1147.1	956.2		
CD4-M4	2550.0	1275.7	851.0	638.3	510.8			
CD4-M5	2896.4	1448.4	966.1	725.1	580.2			
CD4-M6	2707.14	1354.6	903.4	677.8	542.9			
CD4-M7	2896.4	1448.6	966.3	725.1	580.4			

### Binding assays

Direct ELISA (Figure 3): The following buffers were used: Coupling buffer: 0.01 M KCl; blocking buffer: 5% BSA in 0.1 M phosphate buffer pH 7.2; sample buffer: 0.1% BSA and 0.01% Tween 20 in 0.1 M phosphate buffer pH 7.2; washing buffer: phosphate buffer pH 7.2 containing 0.01% Tween 20. Antibody buffer: Phosphate buffer pH 7.2 containing 1% BSA and 0.1% Tween 20. The wells of 96-well Ni-Chelate plates from Thermo Scientific were coated overnight at 4 °C with peptide solution (100 µL, 4 µM in coupling buffer). After unspecific binding had been blocked with a blocking buffer (1 h at room temperature, gentle agitation), the plates were washed twice. Plates were then incubated for 2 h with 100 µL/well gp120(IIIB) from ImmunoDiagnostics at 0.25 µg/mL (Figure 3, left), or in serial dilutions starting at 2 µg/mL (Figure 3, right) in sample buffer, and washed four times. Following incubation with 100 µL/well sheep-anti-HIV-1-gp120 (D7324) from Aalto Bio Reagents (1:5000 in antibody buffer) for 1 h at room temperature, plates were washed four times with washing buffer, incubated for 1 h at room temperature with 100 µL/well rabbit-anti-sheep-IgG peroxidase conjugate from Dianova (diluted 1:5000 with antibody buffer), and washed four times with washing buffer. Plates were developed with 100 µL/well substrate solution (1 mg/mL OPD in 0.03% H_2_O_2_/H_2_O) in the dark (approx. 20 min), and the reaction was stopped by addition of 2 M sulfuric acid (50 µL/well). Absorbances (*A*) were read at 492 nm by using a multichannel photometer (Infinite F200 from Tecan).

Relative Affinities (*A*_r_) were calculated according to the following formula:

*A*_r_ = (*A*_peptide_ – *A*_blank_) / (*A*_1_– *A*_blank_)

in which *A*_1_ is the absorbance of a well coated with CD4-M1, and *A*_blank_ is the absorbance of a well without any peptide.

Enhancement Assay (Figure 4): The following buffers were used: Coating buffer: 0.1 M Na_2_CO_3_, pH 9.5; blocking buffer: 1% BSA in 0.1 M phosphate buffer pH 7.2; sample buffer: 0.1% BSA and 0.01% Tween 20 in 0.1 M phosphate buffer pH 7.2; washing buffer: phosphate buffer pH 7.2 containing 0.01% Tween 20. The wells of 96-well Immulon 2HB plates from Thermo Labsystems were coated overnight at 4 °C with 100 µL mAb X5 (0.25 µg/mL in coating buffer). After unspecific binding had been blocked with blocking buffer (1 h at room temperature), the plates were washed twice. CD4-M5 (50 µL, 60 µM) or sCD4 from SinoBiologicals (50 µL, 0.4 µg/mL) was pre-incubated with gp120(MN) from Immune Technology (50 µL, 0.5 µg/mL) for 10 min. Then the pre-incubated mixture of gp120(MN) with CD4-M5 or sCD4 was added to the X5-coated plate, incubated for 2 h, washed four times, and developed as described above. Absorbances (*A*) were read at 492 nm, and corrected for the respective blanks (wells without X5). All binding assays were performed at least twice, each time in duplicate.

### MD simulation

Computational analysis included modelling of peptide–gp120 complexes followed by molecular dynamics simulation to study the stability and dynamics of the respective complexes. Three gp120-complexes were investigated: gp120 in complex with CD4-M1 and CD4-M2, as well as with CD4. All protein structures were derived from the crystal structure [3], which was treated as explained in the following. First, the gp120-CD4 part was extracted and the nonresolved V4 loop of gp120 was added by using SwissModel [26]. Shortened loops V1, V2 and V3 of gp120 were treated according to the crystal structure. This procedure yielded the setup structure for the full length CD4–gp120 complex, from which all CD4 residues except 22–64 were deleted to obtain the starting structure for the linear peptide CD4-M1. To obtain the complex for CD4-M2, mutations of S23C and D63C and the connecting disulfide bridge were additionally introduced by using Sybyl 7.3 [27]. Final system preparation and MD simulation were carried out according to standard approaches [22,28] by using the software package AMBER [29–31] with the ff99SB force field [32]. Following neutralization with an appropriate number of chloride ions, each protein complex was placed in a standard TIP3P water box [33] with at least 12 Å space to the box boundaries. Energy minimization, heating and equilibration were carried out as explained previously [22]. Subsequently, all complexes were subjected to 100 ns simulation at 310 K with boundary conditions and a 2 fs time step by using SHAKE [34]. Analysis was based on a total of 1000 structures taken from the final 20 ns of the MD simulation at an interval of 20 ps.
